# Reconstruction of mammalian oocytes by germinal vesicle transfer: A systematic review

**Published:** 2017-10

**Authors:** Sara Darbandi, Mahsa Darbandi, Hamid Reza Khorram Khorshid, Abolfazl Shirazi, Mohammad Reza Sadeghi, Ashok Agarwal, Safaa Al-Hasani, Mohammad Mehdi Naderi, Ahmet Ayaz, Mohammad Mehdi Akhondi

**Affiliations:** 1 *Reproductive Biotechnology Research Center, Avicenna Research Institute, ACECR, Tehran, Iran. *; 2 *Genetics Research Center, University of Social Welfare and Rehabilitation Sciences, Tehran, Iran.*; 3 *Center for Reproductive Medicine, Glickman Urological and Kidney Institute, Cleveland Clinic, Cleveland, OH, USA. *; 4 *Reproductive Medicine Unit, University of Schleswig-Holstein, Luebeck, Germany. *; 5 *Yildiz Technical University, Istanbul, Turkey.*

**Keywords:** Germinal vesicle, Micromanipulation, Nuclear transfer, Oocyte

## Abstract

Nuclear transfer procedures have been recently applied for clinical and research targets as a novel assisted reproductive technique and were used for increasing the oocyte activity during its growth and maturation. In this review, we summarized the nuclear transfer technique for germinal vesicle stage oocytes to reconstruct the maturation of them. Our study covered publications between 1966 and August 2017. In result utilized germinal vesicle transfer techniques, fusion, and fertilization survival rate on five different mammalian species are discussed, regarding their potential clinical application. It seems that with a study on this method, there is real hope for effective treatments of old oocytes or oocytes containing mitochondrial problems in the near future.

## Introduction

Fully grown mammalian oocytes can be arrested at the G2/M boundary of the meiosis I (MI) division with a very large nucleus called the germinal vesicle (GV) for months. In the human ovary, each oocyte restarts maturation and meiosis division in reaction to gonadotrophins. In this condition, their GV break down (GVBD) and chromosomes condense. Then the chromosomes arranged in MI stage and oocytes are arrested in MII, ready for fertilization. Preparing the GV oocytes for development to MII maturation is needed many biological factors (1-3).

Both nuclear and cytoplasmic maturation are considered important for normal oocyte development (1-3). The ooplasmic organelles specially mitochondria contribute to the quality of oocytes and probably play an important role in the maturation, fertilization, implantation and embryo development process (3). Therefore a large number of GV oocytes cannot become mature due to defects in their ooplasm and mitochondrial function (3).

Nuclear transfer (NT) is one of the most widely used techniques that seemingly be helpful to improve the ooplasm quality and increase the mitochondria activity through removing the chromosomes from oocyte and transferred into enucleated donated one (3-5). Germinal vesicle transfer (GVT) is possible to enucleate GV stage oocytes before they mature to the MII stage and use them as recipients for NT. 

Although it has been shown that the GVT yields are much less than the two other methods of NT, because of the need to manipulate the oocyte in the early stages of development, in this review we summarized this novel technique for mammalian oocytes. To the hope that by focusing on this method can be found an appropriate way to manipulate immature human oocytes. This work evaluated and compared all designed and optimized mammalian GVT methods to see the best results of other investigator and predict the best method for each probable application. Also, GVT process (including oocytes collection, cell preparation, micromanipulation, electrofusion, and fertilization of the reconstructed oocytes) were summarized that apparently are helpful to improve ooplasm quality and increase the activity and the copy number of mitochondria (3). At the end of each section, the comparison table of embryonic development potential after GVT is provided (5, 6).

## Materials and methods

**Figure 1:**
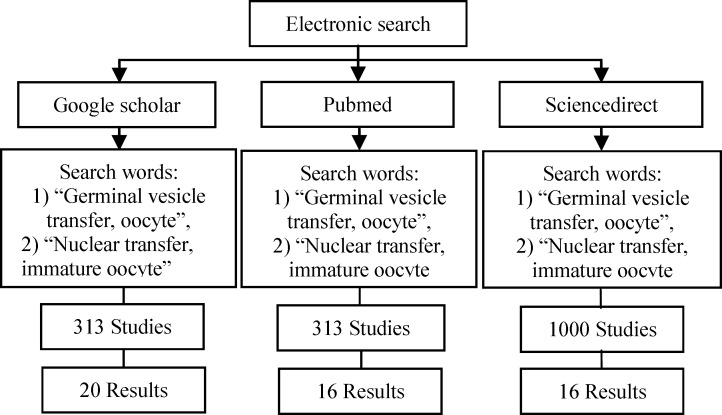
Literature search; the search covered the years 1966 to August 2017. A total of 18,901 articles reviewed, 137 articles were selected. Among these 137 selected articles which one was irrelevant, duplicate or publication with duplicate data was excluded, so 52 articles remained.

The literature search was based on an electronic search using PubMed, Science Direct, and Google Scholar. Initially including keywords were “germinal vesicle, nuclear transfer, oocyte”, “nuclear transfer, immature oocyte” (Figure 1). The searches covered the years between 1966 and August 2017 and contained English publications, other language article abstracts, and these article references. Among these selected articles, which were irrelevant, duplicate or publications with duplicate data were excluded. 

## Results

Of these articles, we investigated all the methods and dedicated equipment have been employed for GVT techniques in mammalian oocytes and followed fusions and fertilization survival rate which are described in details. Procedures have been performed on five different mammalian species (including Murine, Rabbit, Bovine, Porcine and Human). This method is used in human oocytes recently, however, the results from animals indicated that GVT is technically possible with a higher rate of early maturation (5, 6).

## Discussion

Micromanipulation procedures were carried out on a warm stage placed on a micromanipulator system including an inverted light microscope, micro-pipette injector/puller and holder, gas pressure regulator, glass capillary tubing, micrometre syringe, manipulation chambers, and anti-vibration table (4, 7-12). The system could be supplemented with a specimen incubator, charge-coupled device camera, shutter controller, epi-ﬂuorescence, digital image processing software, and computer (7-10, 13-16). Micro-pipettes were made of borosilicate glass (4, 7-10). Electrocell manipulator and the piezoelectric actuator have been respectively used for electrofusion and intracytoplasmic sperm injection (ICSI) (7, 8, 10-12, 15-31). Modular incubator with 5% CO_2_/5% O_2_/ balance N_2_, and 5% CO_2_/21% O_2_/ balance N_2_ are essential for the process (7-10, 15, 16). The procedure is including immature oocytes collection, cell preparation, micromanipulation, electrofusion, and fertilization in five different mammalian species which explained separately in following parts:

**In mouse**

**Immature oocytes collection**

The GV stage immature oocytes could be collected by puncturing ovarian follicles after the one of these stimulating methods including follicle stimulating hormone (FSH) (44-48 h post-injection) (4), 5IU equine chorionic gonadotropin (eCG) (36-48 hr post-injection) (6, 7, 11, 18, 21, 32), 10 IU eCG 48 hr subsequently 10 IU of human chorionic gonadotropin (hCG) (1-3 hr after hCG injection) (19) or 5, 7.5, 8, or 10 IU of pregnant mare serum gonadotropin (PMSG) (46-48 hr post-injection) (9, 12, 20, 22, 23, 33). Then cumulus cell-oocyte complexes (COCs) were released using a fine gauge needle or forceps and were collected with medium (HTF) supplemented with 10% fetal calf serum (FCS) (21).

**Cell preparation**

Cell preparation step consists of two phases

A) Cumulus–corona cells (CCs) removing: CCs of all oocytes have been removed either chemically by a brief exposure to one of these media: medium (M2) containing 500 IU/ml hyaluronidase (Hya) (23) or serum-free medium (HTF) containing 300 IU/ml Hya (18, 19) or mechanically by repetitious pipetting (4, 7, 10-12, 20, 32). Some researchers stated that before oocyte stripping, COCs should be released into one of these supplemented media for 1-2 hr (7, 9, 11, 20); HEPES [4-(2-Hydroxyethyl) -1-piperazineethanesulfonic acid]-medium (M2) with dibutyryl cyclic AMP (dbcAMP) (9), medium (M2) with 0.2 mM 3-isobutyl-1-methylxanthine (IBMX) as collection media and medium (MEM-a) with 20% fetal bovine serum (FBS) and 0.2 mM IBMX, as culture media (7, 11) and medium (HTF) with 10% (v/v) FBS and 0.1 mM IBMX (20).

B) Immature oocytes culturing: Immature naked oocytes were cultured in another medium to preparing oocyte for manipulation or removing whole zona pellucida (2, 4, 6, 7, 9, 10, 12, 14, 18, 19, 23). It was shown that zona-free GV oocytes can also be used as a GV donor (7, 23). Some research groups have used only one type of supplemented media to culture cumulus-free and zona free GV oocytes 1/2-2 h before and during manipulation (7, 32). The methods Including: 1) medium (HTF) with 10% FCS and 50 µg/mL IBMX (32), 2) medium (MEM-a) containing 0.2 µg/ml demecolcine (Dem), 5 µg/ml Cytochalasin B (CB) and 0.2 mM IBMX (7), 3) modiﬁed HTF medium (mHTF) with 10% FCS supplemented with 7.5 g/ml CB at room temperature (33). 

Some others have used two types of supplemented media to culture (2, 4, 9, 18, 19, 34, 35). The methods are: 1) medium (HTF) containing 10% FCS, 50 μg/ml IBMX and 7.5 µg/mL CB at room temperature (RT) for 1/2 hr and then medium (HTF) containing 10% FCS and 50 µg/mL IBMX 4-6 h before and during manipulation (18, 19), 2) medium (M199) with 10% FBS for 2 and 5 h to receive prometaphase I (ProMI) and MI oocytes, respectively (2, 9, 36) and then medium (M2) with 10 μg/ml CB, 0.25 μg/ml nocodazole and 0.2 mM dbcAMP in a micromanipulation chamber (9, 34, 35). 3) IBMX medium containing 5 μg/ml CB and 0.2 μg/ml Dem for 0.5h (11) and then medium (M2) containing 0.2 mM IBMX, 5 μg/ml CB, and 0.2 μg/ml Dem for micromanipulation (11). 4) medium (HTF) with 10% FCS and 50 µg/mL IBMX for 6h incubation and modiﬁed medium (mHTF) with 10% FCS supplemented with 7.5 g/ml CB for 15 min at room temperature to interrupt microﬁlament and rise plasma membrane ﬂexibility before manipulation (33), 5) medium (M2) complemented with 2.5 μ M milrinone for 2 hr to keep the oocytes at the GV stage during micro-manipulation and manipulation drops of medium (M2) with 2.5 μ M milrinone, 5 μ g/ml CB and 0.2 mg/ml Dem (37). 

Others have used three types of supplemented media (4, 6, 10, 12, 14, 23) with these methods: 1) 0.5% pronase in PBS, 10min to removing ZP then medium (M2) with 5 mg/ml CD and 3 mg/ml nocodazole, 30 min (6) and medium (M2) with 150 mg/ml dbcAMP at 37oC in air for all of the manipulations (6). 2) HEPES-medium (KSOM) with 0.2 mM IBMX, 2h before manipulation then HEPES-medium (KSOM) with 2 μg/ml CD and 1 μg/ml nocodazole, 15 min at RT(4) and 5 µl droplets of medium (M2) with 3mg/ml BSA and 25 µg/ml CB for micromanipulation (4). 3) medium (M199) with 0.2 mM IBMX, ~2h (10) or medium (Waymouth) with 0.2 mM IBMX, 0.23 μM Pyruvate and 5% FBS until the GVT procedure (12, 14) then at least 15 min in medium (M2) with 25 µg/ml CB and 3 mg/ml bovine serum albumin (BSA) (10, 12) and 5 µl droplets of medium (M2) containing 3 mg/ml BSA and 25 µg/ml CB for manipulation (10, 12). 4) medium (M199) containing 10% FCS and 20 µg/ml IBMX, 2h (23) then medium (M119) with 0.3% pronase, 3-4 min (23) and IBMX-containing medium for maintaining until injection (23). 

**Micromanipulation**

Micromanipulation can be done in two developmental stages of oocyte including prophase1 and metaphase1 (M1) of meiosis. To imagine the nuclear material (in prophase1oocytes) and meiotic spindle (in MI oocytes) localization, oocytes could be incubated in the enucleating medium with or without 2% sucrose for 1/2 hr (9, 37, 38). Indirect enucleation (9, 10, 18, 23), direct enucleation (4, 7, 9-12, 17-21, 32, 39, 40) and oocyte fraction (6, 38) are three enucleation methods for GV oocytes. In the first method, enucleation was performed ‘indirectly’, to eject karyoplast via a slot made in the ZP by rising inside holding pipette pressure (9, 10, 18, 23). GV- or MI-oocyte enucleation was accomplished using this way (9, 40). 

The enucleation pipette with inner diameter (ID) of 10-15 µm (9, 23) or 20-25 µm was inserted moderately into the perivitelline space (PVS) via the ZP (4, 7, 10-12, 17-21, 32, 39) ProMI- or MI-karyoplast was aspirated with the smallest possible volume of surrounding ooplasm by smooth suction and the pipette was moderately withdraw from the oocyte (9). Then karyoplast was inserted inside the PVS of enucleated oocyte against the holding pipette (4, 7, 10-12, 17-21, 32, 39) through the slit was made in the ZP with the same pipette (9, 40) or another pipette (ID: 25µm) (22, 23). In the second method, to speed removing and replacing the GV karyoplast and determine the size of it, the process was done ‘directly’ by a tapered enucleation pipette with an inner diameter of about 20 μm (4, 7, 9-12, 17-21, 32, 33, 37, 39, 40). Also, GVs and karyoplasts can be located in the milrinone-medium (M16) for additional manipulation (37).

Afterward these two methods, the reconstructed oocytes could be rinsed in enucleation media and incubated in medium (M2) containing dbcAMP (9) or in medium (M2 or HTF) at 37^o^C, 5% CO_2_ for 15 or 30 min until electrofusion (21, 32). Or equilibrated in medium (M199) with 10% FCS for 30 min (23) or in medium (MEM-α) with 0.2 mM IBMX for 1 hr (7). In these two methods the enucleated oocytes could be stained with 10 µg/ml Hoechst33342 (H342) for 8-10 sec and evaluated by fluorescence microscopy (18, 23, 41). To reduce sticking of the membranes within the pipet, the micropipettes could be rinsed in 10% polyvinyl pyrrolidone (PVP) before GV removal and then two or three droplets of mineral oil might be aspirated to control the fluid flow during the manipulation (7, 37). In the third method, the oocytes were extended in a very narrow pipette and splitted precisely into 4 equal pieces of oocytes and GV karyoplast (6, 38). The GV karyoplast was rinsed in medium without dbcAMP then cultured in medium (M199) containing 0.2 mM Na-pyruvate, 25 µg/ml gentamicin and 4 mg/mL BSA for 2-3h in a 5% CO_2_ and 37^o^C before fusion (6, 38). Also, some ooplast remained in the medium containing dbcAMP before fusion (6, 38). 

GV karyoplasts were rinsed a few times in medium (M2) then both components (GV karyoplast and ooplast) were incubated in PBS with 200 µg/ml phytohemagglutinin (PHA) (6, 38). The Proximity interactions among these both components were obtained by pipetting with a narrow pipette (6, 38). These ingredients were incubated in medium (M199) containing 1 g/ml polyethylene glycol (PEG) for 50-55 sec (6). The reconstructed oocytes were rinsed in medium (M2) then cultured in medium (M199) with dbcAMP for 30 min and moved into dbcAMP-free medium (M199) to GVBD and maturation (6). The reconstructed oocytes were inspected periodically (15, 30, 60 min, 3 and 12 hr) (6). 

**Electrofusion**

Electrolyte medium and non-electrolyte medium are two types of electrofusion medium poured among two parallel-electrodes jointed to the generator (17, 20, 21). Non-electrolytic medium was 0.25M sucrose or combination of 0.28 M sucrose, 0.5 mM Magnesium Acetate, 0.1 mM Calcium Acetate, 0.1 mM Potassium phosphate dibasic, 0.1 mM glutathione, and 0.01 mg/ml BSA (10, 12, 14) with final osmolarity 280 mOsm/l and pH 7.3 (10, 12, 14, 32). Electrolyte medium usually is combination of 0.27-0.3M mannitol, 0.05-0.1 mM Calcium chloride, and 0.05-0.1 mM Magnesium sulfate in medium (M2 or HTF), or PBS with or without 0.3% BSA (9, 17-21, 23, 33). It seems that the electrolyte medium works much better than the non-electrolyte (10, 14). After manual alignmen (7, 9-12, 14, 23, 32) or alignment with brief application of a low-voltage alternating current (AC) pulse (0.1-0.3 kV/cm 2 MH for 5-10 sec or 6-8 V for 5-10 sec) (17-21, 33), one of these fusion pulses were used; single or double direct current (DC) pulses (1.0-2.5 kV/cm DC for 50-90 µs) (9, 10, 12, 14, 17-21, 32), three DC pulses (1.8-2.5 kV/cm DC for 50-80 µs) (23, 33), or single DC pulse (0.9 kV/cm for 10 µs) (7, 11).

The incorporation in reconstructed oocytes was monitored 10, 20 and 30min after each electropulse (10, 12, 14, 21, 23, 32). Fusion usually occurs within 40-60 min (17-21). After that, the reconstructed oocytes were washed three times in one of these three types of media; supplemented medium (M2) with 0.2 mM dbcAMP at 37^o^C (9), medium (M2, HTF or MEM- α) containing 20% FBS (7, 11, 17-21), IBMX free supplemented medium (M16, HTF or Waymouth) with 10% FCS at 37^o^C, 5% CO_2_ (10, 12, 14, 32, 33, 37). 

For in vitro maturation (IVM), reconstructed oocytes could be placed in one of these media; IBMX free supplemented medium (M16, HTF, M199 and Waymouth) with 10% FCS with or without 75 μg/ml penicillin G potassium salt (Pen-GK) and 50 μg/ml streptomycin sulfate (Str-S) at 37^o^C, 5% CO_2_ for 14-16hr (9, 10, 12, 14, 17-21, 32), supplemented medium (M199) with PMSG for 24 hr (23), and medium (MEM- α) containing 20% FBS up to 14 hr proceeding to the GV, GVBD, MI and MII stages (7, 11). Instead of electrofusion, Sendai virus (SeV) could be accomplished too. 

The GV karyoplast from a donor oocyte was aspirated inside a pipette then instantly inactivated SeV from its droplet with the volume of the two-thirds of aspirated GV karyoplast content was extracted to this pipette (4). The GV karyoplast and the inactivated SeV were released together inside the PVS of an enucleated ooplast (4). Reconstructed oocytes fusion was done within 30-60 min without any electrofusion (4). Afterward, these oocytes were washed briefly in HEPES-medium (KSOM) (4). 

**Fertilization**

According to the mentioned articles, fertilization was done by using one of the following methods.

ICSI: ICSI was done in HEPES-CZB within 1-2 hr after NT (4, 11, 12, 42). Immobilized spermatozoa were injected inside the ooplasm in HEPES-medium (KSOM) containing 20% FBS (4, 7, 8, 11, 12, 29-31, 42). In this way, a small droplet of sperm suspension incubated for 1 hr at 37^o^C was mixed thoroughly with an equal volume of HEPES-CZB medium with 12% (w/v) PVP immediately before ICSI (7, 8, 11, 12, 29-31). A holding pipette kept the MII oocyte and a sperm head was aspirated into an injecting pipette (4, 7, 8, 31). Then the injecting pipette was penetrated through the ZP with multiple piezoelectric pulses and oolemma was pushed into the oocyte with a single piezoelectric pulse (4, 7, 8, 31). The needle without damaging the oolemma was slowly withdrawn (4, 7, 8, 31). All embryos were cultured in the medium (CZB, KSOM or HTF) under 5% CO_2_ and 21% O_2_ in nitrogen at 37^o^C (11, 20). The oocytes were examined within 5-6h to observe pronuclei (PN) and a clear second polar body (PB) (11, 12, 20, 29, 30), 24 hr (to observe 2-cell embryo), 48 hr (to observe 3-cell or 4-cell embryo), 72 hr (to observe morula or early blastocyst) and 96h (to observe blastocyst) for evaluation of the embryo development (4). 

In vitro fertilization (IVF): The MII oocytes were fertilized in droplets containing sperm and IVF medium (HTF) (9). Prior to fertilization, the ZP was completely removed (zona-free oocytes) or it was partially gapped (zona drilled oocytes) in a Tyrode’s solution with pH 2.5 containing PVP (9). For fertilization, zona-free oocyte was 2 hr incubated in 50 motile sperms /droplet and zona-drilled oocyte was 4 hr incubated in 0.5×10^6^ motile sperms/ml. After fertilization, oocytes were rinsed and then cultured in equilibrated medium (KSOM) for further culture (37^o^C, 5% CO_2_) (9).

4.1.6. Comparing the potential of embryonic development after GVT As a summary, 14 papers demonstrated only survival rate of reconstructed oocytes and 10 papers indicated the survival rate of both reconstructed oocytes and embryos according to papers shown in these several methods (Table I).

**Table I:** Comparing the potential of embryonic development after GV nuclear transfer (GVT) in murine sample

**Paper**	**Cell used for GVTT (N)**	**Successful GVTT (N)**	**Successful fusion** **n (%)**	**Survival after fussion n (%)**	**Survival after fertilization or activation n (%)**	**Two-cell embryo** **n (%)**	**Blastosyst n (%)**
Neupane *et al* (4)	41	39	35 (90)	25 (71)	14 (56)	13 (93)	0
Fulka *et al* (6)	350	272	195 (72)	45(23)	-	-	-
Mohammed *et al* (9)	CE: 35		23 (66)	22 (94)	-	-	-
	SE: 30		21 (70)	20 (93)	-	-	-
	ProMI: 35		25 (70)	15 (60)	-	-	-
	MI: 47		34 (72)	14 (40)	-	-	-
Takeuchi *et al* (10)	45	42	39 (93)	36 (92)			
Cheng *et al* (11)	DBD		1419 (64)	901 (63)	771 (86)	-	427 (55)
	BBD		1262 (89)	1119 (89)	627 (56)	-	346 (55)
Takeuchi *et al* (12)					122	80 (66)	70 (57)
Takeuchi *et al* (14)	-	53	45 (84.9)	43 (95)	52 (65.8)	51 (98)	11 (21)
Wang *et al* (17)		101	36 (36)	19 (53)	19 (100)	-	-
Liu *et al* (18)	Auto: 88	81	70 (86)	32 (46)	-	-	-
	Hetero: 196	185	144 (78)	95 (66)	-	-	-
Moffa *et al* (21)	FF: 170	135	55 (41)	44 (80)	-	-	-
	TT: 162	138	57 (41)	52 (91)	-	-	-
	FT: 169	143	73 (51)	63 (86)	-	-	-
	TF: 156	124	49 (40)	38 (78)	-	-	-
Li *et al* (23)	71	-	-	51 (72)	32 (63)	4 (12)	2 (6.2)
Cui *et al* (32)	YY	353	317 (90)	305 (96) A: 157-I: 147	A: 134 (85) I: 124 (84)	A: 87 (65) I: 66 (53)	-
	YA	179	169 (94)	158 (93) A: 85-I: 73	A: 69 (81) I: 60 (82)	A: 48 (70) I: 32 (53)	-
	AY	200	187 (94)	183 (99) A: 102-I: 81	A: 80 (78) I: 65 (80)	A: 57 (71) I: 44 (68)	-
Zhang *et al* (33)	GV/C	52	52 (100)	51 (98)	-	-	-
	GV/C ^IBMX6h^	37	37 (100)	16 (43)	-	-	-
	GV ^IBMX6h^/C	35	35 (100)	35 (100)	-	-	-
Wang *et al* (37)	GV ^m^/C^m^	-	-	-	-	-	-

CE: Indirect enucleated oocytes

SE: Direct enucleated oocytes

DBD: D2 ooplasm/ B6 GV donor/ D2 ICSI spermatozoa

BBD: B6 ooplasm/ B6 GV donor/ D2 ICSI spermatozoa

YY: Younged-Younged

YA: Younged-Aged

AY: Aged–Younged

FF: Fresh GV-Fresh cytoplast

TT: Thawed GV-thawed cytoplast

FT: Fresh GV-thawed cytoplast

TF: Thawed GV-fresh cytoplast

A: Activated oocytes

ProMI: Prophase mitosis I

I: In vitro fertilizated oocytes

MI: Metaphase I

GV/C: Non-IBMX-arrested GV/non-IBMX-arrested cytoplasm

GVm/Cm: Mouse GV/mouse cytoplasm

GV/C^IBMX6h^: Non-IBMX-arrested GV/IBMX-arrested cytoplasm

GV^IBMX6h^/C: IBMX-arrested GV/non-IBMX-arrested cytoplasm

**In rabbit**

**Immature oocytes collection**

The female rabbits were received intraperitoneal injections of 120-150 IU PMSG and killed 72 hr later. COCs were aspirated from excised ovaries (23). COCs also could be administered by intramuscular injection of 0.8-1.0 mg/per rabbit FSH twice daily for 3 days (22).

**Cell preparation**

COCs were denuded mechanically by exposing to medium (M2) with 500 IU/ml Hya for about 5 min (22, 23). Completely denudation of all rabbit COCs was difficult, so partially denuded oocytes were moved to medium (M199) containing 10% FCS and 20 µg/ml IBMX for 1.5-2 hr (22, 23) and cultured for 30 min in medium (M2) supplemented with 10% FCS, 7.5 µg/ml CB, and 20 µg/ml IBMX at RT before enucleating the cell (22, 23).

**Micromanipulation**

The injecting pipette (ID: 25-30µm) penetrated inside the PVS versus the holding pipette via the cut made in the ZP and removed the GV karyoplast (10, 18, 22, 23). The diameter of the GV karyoplast was about 30-40 µm (10, 18, 22, 23). The enucleation was performed indirectly through increasing the inside holding pipette pressure (10, 18, 22, 23). Then the reconstructed oocytes were transferred to medium (M199) supplemented with 10% FCS for 1/2 hr (22, 23).

**Electrofusion**

The reconstructed oocytes were moved into a droplet of fusion medium containing 0.3 M mannitol, 0.1 mM calcium chloride and 0.05 mM magnesium sulfate and aligned with 2 DC pulses (1.8 kV/cm for 80 µs) (22). About 30 min later, the fused reconstructed oocytes were moved to culture medium (M199) containing PMSG, 10% FCS or 10 IU/mL eCG for 18-24 hr at 38^o^C and 5% CO_2_ (22, 23).

**Fertilization**

ICSI: Spermatozoa was washed and suspended for 1h in 1.5 ml medium (M2) (22, 23). Spermatozoa (300 gr, 5 min) was centrifuged and resuspended in medium (M2) with 10% PVP (1:1) (22, 23). The injection needle had 6-7µm inner and 8-9 µm outer diameter (OD) (22, 23). Immediately after ooplasmic injection, the injection pipette was drown back quickly and the oocyte was liberated (22, 23). Following, the oocytes were transferred into maturation medium (RPMI 1640:M199=1:1) with 10% FCS or medium (M199) at 37.5-38^o^C and 5% CO_2_ and examined every 24 hr (22, 23).

Comparing the potential of embryonic development after GVT in several mentioned methods. 

For conclusion, one paper demonstrated survival rate of reconstructed oocytes and 4 papers showed the survival rate of both reconstructed oocytes and embryos (Table II).

**Table II:** Comparing the potential of embryonic development after GV nuclear transfer (GVT) in rabbit sample

**Paper**	**Cell used for GVT (N)**	**Successful GVT (N)**	**Successful fusion**	**Survival after fusion**	**Survival after fertilization or activation**	**Two-cell embryo **	**Blastocyst **
Li *et al* (22)	WW: 115	-	92 (80)	74 (80)	53 (72)	31 (58)	5 (9.4)
	BB: 43	-	37 (86)	25 (68)	17 (68)	11 (65)	1 (5.9)
	WB: 114	-	101 (89)	79 (78)	56 (71)	34 (61)	4 (7.1)
	BW: 79	-	68 (86)	55 (81)	41 (75)	23 (56)	3 (7.3)
Li *et al* (22)	201	112	61 (54)	47 (77)	-	-	-

Data presented as n (%).

W: white rabbit

B: indigo blue rabbit

GVT: germinal vesicle transfer

**In cattle**

**Immature oocytes collection**

Ovaries were derived from the slaughterhouse and they transferred to the laboratory in sterile isotonic saline within 2 hr at 26^o^C or within 1 hr at 20^o^C (15, 16, 43). COCs were restored with a 16-gauge needle connected to a vacuum pump with a 28 mm/Hg suction pressure (15, 16, 25). The COCs were washed in supplemented medium (M199) with 20 mM HEPES, 1,790 U/l heparin, and 0.4% BSA (15, 16) or in modified PBS and cultured in medium (M199) containing 5% (v/v) BCS, 100 U/ml Pen-GK and 50 µg/ml Str-S at 39^o^C, 5% CO_2_ and 95% air (15, 16, 25). 

**Cell preparation**

CCs were removed mechanically using vortex agitation (2 min, 35 Hz) in medium (M199) containing 20 mM HEPES and 5% FCS (15, 16) or chemically using Hya (300 IU/ml) (24). Denuded oocytes were transferred to medium (M199) containing 10% FCS and 0.2 mM IBMX (15, 16, 44). For partially removing ooplasmic lipid droplets and visualizing the nuclear envelope, a group of 10-15 oocytes were centrifuged (5 min at 17000 g) 30-40 min prior to each micromanipulation, and were cultured in medium (M199) containing 10% FCS and 7.5 μg/mL CB for 30 min (10, 15, 16). To make the plasma membrane more suitable for micromanipulation, the GV oocytes could be treated with CB and H342 (5 µg/ml) (24).

**Micromanipulation**

Micromanipulation was performed in medium (M199) containing 0.2 mM IBMX, 20 mM HEPES, and 10% FCS (15, 16). The GV envelope was identified using carefully rotating the oocyte then the ZP was lanced immediately overlying the GV with a tapered micropipette. The GV karyoplast was removed with 40-45 µm in diameter (10, 15, 16). Some investigators who had not added H342 stain to CB in the previous step stained the oocytes in medium (M199) containing 1µg/ml H342 in darkness for 5 min to visualize the chromatin in the GVs (15, 16). Karyoplast membrane was allowed to settle for 10 min following the enucleation procedure (10, 15, 16). The remaining ooplast could be placed into a small droplet of medium (M2) containing 5 mg/ml CB and 0.1 mg/ml colcemid (25, 45-49). 

Each GV karyoplast in a 40% PVP droplet was suctioned inside the injection pipette (ID: 35-40 µm) and inserted to the PVS of another ooplast, via the ZP incision (10, 15, 16). The reconstructed oocytes were transferred to medium (M199) containing 10% FCS, 0.68 mM L-glutamine, 25 mM sodium hydrogen carbonate, and 0.2 mM sodium pyruvate at 38.5^o^C and 5% CO_2_ for 1/2 hr before electrofusion (15, 16).

**Electrofusion**

Some investigators stated that fusion chamber could be filled with 300 μl of a non-electrolytic medium (0.25M sucrose) (10, 15, 16) using double DC pulse (1.0 kV/cm, 70 μs, 1/2 hr intervals) (15, 16). Others stated an electrolytic medium (0.3M mannitol, 0.1 mM calcium chloride and 0.05 mM magnesium sulfate in H_2_O) could be used with a double DC pulse (2 kV/cm, 30 µs, 0.1 sec intervals) (24). After electrofusion, the reconstructed oocytes were cultured in medium (M199) containing 10% FCS, 0.68 mM L-glutamine, 25 mM sodium hydrogen carbonate, 0.2 mM sodium pyruvate, and 0.1 IU/ml of recombinant human FSH (RH-FSH) in attendance of an equal number of unscathed COCs for 24 hr at 38.5^o^C and 5% CO_2_ with the aim of completing the first meiotic division (15, 16). 

Upon attainment of the MII stage, the reconstructed oocytes were assigned to IVF or ICSI (10, 15, 16, 24, 25). Instead of using electrofusion, SeV could be introduced inside the PVS of an enucleated ooplast (25, 45-49). The reconstructed oocytes were transferred to a medium (Weymouth) with dbcAMP (25, 45-49). Fusion usually occurred within 1/2 hr (25, 45-49). The reconstructed oocytes were washed and cultured for 22 hr with CCs, in medium (M199) containing 5% (v/v) FCS, 100 U/ml Pen-GK and 50 µg/ml Str-S in 5% CO_2_ at 39^o^C (25, 45-49).

**Fertilization**

IVF: The reconstructed oocytes were fertilized with sperm suspension in IVF medium containing 5 mM caffeine, 2.5 IU/ml heparin, and 5 mg/ml BSA (25). After 6 hr, the oocytes were transferred into the medium (M199) containing 5% (v/v) FCS, 100 U/ml Pen-GK, 50 μg/ml Str-S, for co-culturing with bovine CCs of fully grown GV oocytes for a period of 8 days (25).

**Comparing the potential of embryonic development after GVT**

For outcome, 4 papers indicated survival rate of reconstructed oocytes and 6 papers indicated the survival rate of both reconstructed oocytes and embryos (Table III).

**Table III:** Comparing the potential of embryonic development after GV nuclear transfer (GVT) in bovine sample

**Paper**	**Cell used for GVT (N)**	**Successful fusion**	**Survival after fusion **	**Two-cell embryo **	**Blastocyst**
Franciosi *et al* (15)	Vitrification: 99	77 (78)	29 (38)	9 (32)	2 (8.1)
	Fresh: 145	101 (70)	42 (42)	28 (67)	13 (31)
Luciano *et al* (16)	Vitrification: 65	47 (72)	18 (38)	-	-
	Fresh: 62	43 (69)	19 (44)	-	-
Consiglio *et al* (24)		(76.34)	(56)	-	(40)
Bao *et al* (25)	I: 74	-	23 (31)	15 (67)	2 (12)
	II: 67	-	27 (40)	17 (62)	2 (11)
	III: 55	-	21 (38)	17 (79)	3 (15)
	IV: 36	-	19 (53)	-	-
	V: 27	-	18 (67)	-	-

Data presented as n (%).

I: 70-90 µm size of donor oocytes.

II: 90-99 µm size of donor oocytes.

III: 100-109 µm size of donor oocytes.

IV: 110-119 µm size of donor oocytes.

V: 120-130 µm size of donor oocytes.

GVT: Germinal vesicle transfer

**In pig**

**Immature oocytes collection**

Pig ovaries were achieved from the slaughterhouse and moved to the laboratory in phosphate buffered saline at 35^o^C within 1 h (37, 50). COCs were aspirated from ovaries with an 18-gauge needle fixed to a 20 ml disposable syringe (37). After three rinses in medium (TCM-199) with 2.2% NaHCO_3_, suitable COCs were selected for culture (37). COCs were cultured in 500 µl medium (NCSU-37) modified by adding 10% (v/v) porcine follicular fluid (pFF), 0.6 mM cysteine, 50mM β-mercaptoethanol, 1mM dbcAMP, 10IU/ml eCG and 10IU/ml hCG for 22 hr. The oocytes were then transferred to modified medium without dbcAMP and hormone and cultured for another 22h in 5% CO_2_, 5%O_2_ and 90% N_2_ at 39^o^C (50). 

**Cell preparation**

Cumulus cells were freed from the COCs after repeated pipetting in 300 IU/ml Hya for 3 min (37) or gentle pipetting in 150 IU/ml Hya (50). Denuded oocytes were cultured in medium (TCM-199) with hormone, 0.1% PVA (w/v), 0.91 mM sodium pyruvate, 75 μ g/ml Pen-GK and 50 μ g/ml Str-S (37). Oocytes were cultured in medium (TCM-199) with 0.1% PVA (w/v), 0.91 mM sodium pyruvate, 75 μ g/ml Pen-GK, 50 μ g/ml Str-S, 0.57 mM cysteine, 0.5 μ g/ml FSH, 0.5 μ g/ml LH, 10 ng/ml EGF and 4 mM hypoxanthine (HX) preventing GVBD (37). Or denuded oocytes were instantly centrifuged in medium (TCM199) supplemented with 5% FBS, 1mM dbcAMP and 5 µM CD at 10,000 g for 10 min at 38^o^C for visualization of the GV (50). 

**Micromanipulation**

Oocytes were exposed to medium (M2) supplemented with 15 μ g/ml CB and 0.1 μ g/ml Dem for 30 min at 38.5^o^C, with 5% CO_2_ in the air (37). GVT was carried out in medium (M2) with 15 μ g/ml CB and 0.1 μ g/ml Dem (37). For enucleation, oocytes were rinsed several times, moved into the medium (M2), and centrifuged for 10 min at 4000 g for visualization of the GV (37). In another protocol without centrifuging, oocytes were exposed to medium (TCM) with 5% FBS, 1mM dbcAMP and 5 µg/ml CD (50), then a slit was made in the ZP by pressing a sharp and thin needle through the PVS against the holding pipette near the place of the GV (50). The GV was then pressed out by pushing on the ZP (37). Oocytes were cautiously pipetted to separate GV from the oocyte (37). Transferring the GV into enucleated oocyte was done either indirectly by moving it into the PVS using a blunt-tip micropipette (ID: 30 μm) in medium(TCM199) with 5% FBS, 1mM dbcAMP and 300 µg/ml phytohemagglutinin (50) or directly by injecting it into ooplasm using a piezo-actuated micromanipulator (37). Indirect transferring, first, a single weak piezo pulse was used to interrupt the oolema around the GV. The pipette (ID: 20 μm) was injected from the slit of the ZP and moved until it reached the opposite side of the oocyte (37). One weak piezo pulse was used to incise the oolemma at the pipette tip, and GV was directly ejected into oolema (37). 

**Electrofusion**

The reconstructed oocyte was moved to a drop of fusion medium (0.3 M mannitol, 0.1 mM CaCl_2_ and 0.05 mM MgSO4 and 0.5 HEPES in sterile water) (50). Electro-fusion was done with 160 V/ mm DC for 60 ms or 2.0 kv/cm for 20 µsec (50) delivered by an Electro Cell manipulator or electroporator. The fusion rate was examined 30 min later (37). Oocytes were incubated in 500 μl medium free of hormones with or without 1mM dbcAMP covered for up to 44 hr in 5% CO_2_ and 38.5 °C (37).

**Fertilization**

IVF: After 44 hr of maturation, reconstructed oocytes were moved into 100-µl droplets of fertilization medium consisted of 90 mM NaCl, 12 mM KCl, 25 mM NaHCO_3_, 0.5 mM NaH_2_PO_4_, 0.5 mM MgSO_4_, 10 mM sodium lactate, 10 mM HEPES, 8 mM CaCl_2_, 2 mM sodium pyruvate, 2 mM caffeine and 5mg/ml BSA covered with oil (50). The concentration of spermatozoa was 1×10^5^/ml oocytes and spermatozoa were co-incubated for 3 hr at 38.5^o^C in 5% CO_2_, 5%O_2_ and 90% N_2_ (50). the oocytes were cultured from Day 0 to Day 2, in IVC medium (NCSU-37) modified by the addition of 0.4% (w/v) BSA, 50 μM β-mercaptoethanol, 0.17 mM sodium pyruvate and 2.73 mM sodium lactate and until Day 6 in medium (NCSU-37) supplemented with 5.55 mM glucose in 5% CO_2_, 5% O_2_, and 90% N_2_ at 38.5^o^C (50). 

**Comparing the potential of embryonic development after GVT**

As a summary, one paper demonstrated survival rate of reconstructed oocytes and one paper indicated the survival rate of both reconstructed oocytes and embryos according to papers shown in these several methods (Table IV).

**Table IV:** Comparing the potential of embryonic development after GV nuclear transfer (GVT) in human sample

**Paper**	**Cell used for GVT (N)**	**Successful GVT (N)**	**Successful fusion n (%)**	**Survival after fusion n (%)**	**Survival after fertilization or activation n (%)**	**Two-cell embryo n (%)**	**Blastocyst n (%)**
Wang *et al* (37)	*GV* ^p^ */C* ^m^	**-**	**-**	**-**	**-**	**-**	**-**
	*GV* ^m^ */C* ^p^	**-**	**-**	**-**	**-**	**-**	**-**
Dang-Nguyen *et al* (50)	*GVTR*	100	100 (100)	100 (100)	100 (100)	-	3 (3)

GVT: Germinal vesicle transfer

GV^p^/C^m^: pig GV/mouse cytoplasm

GV^m^/C^p^: Mouse GV/pig cytoplasm

GVTR: Rescued by transferring germinal vesicles (GV) into enucleated cytoplasts

**In Human**

**Immature oocytes collection**

The spare human GV-oocytes were retrieved from women treated using ICSI after controlled ovarian hyperstimulation; either with high purity urinary FSH (U-FSH) or with RH-FSH after pituitary suppression with gonadotropin-releasing hormone (GnRH) agonist and ovary stimulation with gonadotropic hormones (27, 51-53). The initial dose of U-FSH was 225 IU/day and RH-FSH was 200 IU/day in the first three days and 150 IU/day on the fourth day (51, 52). In the fifth day, the daily FSH dose was adjusted every other day based on the serum levels of estradiol (E2) and the volume and number of ovarian follicles (51, 52). When a minimum of three follicles reached 18 mm, 5,000 or 10,000 IU HCG was given to ovulation induction (26, 27, 33, 51, 52). After 36 hr, follicular aspiration was done (33, 51, 52). The immature oocytes which were unsuitable for ICSI were used for NT after obtaining the patient consent (26, 27). 

**Cell preparation**

CCs could be removed enzymatically and mechanically using repeated pipetting in medium (HTF) containing 80 IU/ml Hya or a brief incubation (20-30 sec) at 37^o^C in medium (G-GAMETE) containing 20 IU/ml Hya and then repeated pipetting (26, 27, 52, 53). GV-oocytes were collected in medium (HTF) containing 10% (v/v) FBS (26). To avoid GVBD, these were moved to medium (M199) containing 0.2 mM IBMX (27). Before Micromanipulation, the GV-oocytes were transferred to a medium (HTF) with only 7.5 µg/mL CB for approximately 1h (26, 28). 

**Micromanipulation**

The ZP was lanced immediately overlying the GV (26-28, 41). Following that, the GV karyoplast was removed indirectly by a micropipette (ID: 20 µm) via a slit made in the ZP and then inserted into an enucleated ooplast and rinsed in medium (HTF) (26-28). Some researchers exposed oocytes to 25 µg/ml CB after lancing the ZP (26, 27).

**Electrofusion**

Electrolytic solution was made of 0.3M mannitol, 0.05 mg/mL BSA, 0.1 mM Calcium chloride, and 0.05-0.1 mM Magnesium sulfate (26-28). The reconstructed oocytes was aligned manually (27) or electrically by exposing to AC pulse of 6-8 V for 6-10 sec (26, 28) and fused using a DC pulses (1.36 kV/cm, 30-40 µs or 1.8-2.5 kV/cm,50 μs or 1.0 kV/cm DC, 99-100 μs) (26-28). Routinely, the reconstructed oocyte fusion was performed within 15-30 min (26, 28). Afterward, the reconstructed oocytes could be co-cultured for 36-50 hr with CCs in medium containing 10% FBS, 0.075 IU/ml FSH, 35 ng/mL insulin, and 20% human follicular fluid (v/v) at 37^o^C and 5% CO_2_ (26). About 24, 36, 42 and 50 hr post- Electrofusion, oocytes were checked (26, 27). 

**Fertilization**

ICSI: The oocyte was kept using slight negative pressure in holding pipette and the injection pipette containing sperm was deeply introduced into the ooplasm (54). Maturated oocytes were only injected and after washing, they returned to medium (B2) and stored in the incubator (54). About 16h after ICSI, the oocytes were checked for intactness, fertilization, cleavage and morphological appearance (54). 

**Comparing the potential of embryonic development after GVT**

For the result, only one paper showed the survival rate of reconstructed oocytes and one paper indicated the survival rate of both reconstructed oocytes and embryos (Table V).

**Table V:** Comparing the potential of embryonic development after GV nuclear transfer (GVT) in human sample

**Paper**	**Cell used for GVT (N)**	**Successful GVT (N)**	**Successful fusion **	**Survival after fusion **	**Survival after fertilization or activation **	**Two-cell embryo **	**Blastocyst **
Zhang *et al* (26)	28	19	12 (63)	7 (58)	-	-	-
Tesarik *et al *(52)	With CT: 50	40	40 (100)	29 (73)	23 (79)	-	-
	Without CT: 50	1	1 (100)	1 (100)	1 (100)	-	-
Zhang *et al* (33)	GV^h^/C^m^	25	25 (100)	25 (100)	-	-	-
	GV^m^/C^h^	18	18 (18)	8 (44)	-	-	-

Data presented as n (%).

CT: Cytochalasin treatment

GVT: Germinal vesicle transfer

GV^h^/C^m^: Human GV/mouse cytoplasm

GV^m^/C^h^: Mouse GV/human cytoplasm

## Conclusion

However, the recent GVT techniques in mammalian oocytes are in progress, we still have a poor knowledge of this technique and the resulting embryos as well as those of future disease because of genetic and epigenetic modifications. Therefore, although many NT methods have been proposed for ART, few of them are truly therapeutic importance for immature oocytes. It should be more clarified by other studies and the clinical use of this method requires further studies.

## Conflict of interest

All authors declare no financial or commercial conflict of interest.
